# *ALS1* Deletion Increases the Proportion of Small Cells in a *Candida albicans* Culture Population: Hypothesizing a Novel Role for Als1

**DOI:** 10.3389/fcimb.2022.895068

**Published:** 2022-05-11

**Authors:** Xiaomin Zhao, Soon-Hwan Oh, David A. Coleman, Lois L. Hoyer

**Affiliations:** Department of Pathobiology, College of Veterinary Medicine, University of Illinois at Urbana-Champaign, Urbana, IL, United States

**Keywords:** *Candida albicans*, cell size, agglutinin-like sequence family, germ tube, murine model, disseminated candidiasis

## Abstract

*Candida albicans* Als1 is a large cell-surface glycoprotein most often discussed for its role in mediating ligand-binding and aggregative interactions. Relative to a wild-type control, deletion of *ALS1* produced a strain that showed delayed germ-tube formation and delayed disease progression in a murine model of disseminated candidiasis. Populations of *Δals1/Δals1* cultured cells had a higher proportion of smaller cells compared to wild-type or *ALS1* reintegrant control cultures. The goal of this work was to investigate whether this difference in cell-size distributions was responsible for delayed germ-tube formation and delayed disease progression. Flow cytometry was used to select populations of wild-type and *Δals1/Δals1* cells with varied cell-size distributions. Delayed germ-tube formation was demonstrated for small cells sorted from a wild-type (*ALS1/ALS1*) culture population. Large cells sorted from a *Δals1/Δals1* culture formed germ tubes as quickly as the wild-type control demonstrating clearly that the *Δals1/Δals1* germ-tube formation delays were attributable to cell size. *In vivo*, smaller-sized cells of the wild-type control showed fewer colony-forming units (cfu) per gram of kidney tissue and less-severe histopathology lesions compared to larger cells of the same strain. The *Δals1/Δals1* strain showed reduced cfu/g of kidney tissue and less-severe lesions compared to the wild-type control. However, isolation and testing of the larger cells from the *Δals1/Δals1* population increased cfu/g of tissue and showed increased lesion severity compared to the overall mutant cell population. *In vivo* hypha lengths from the large, sorted *Δals1/Δals1* cells were comparable to those for the wild-type control strain. These results demonstrated that a large share of the *Δals1/Δals1 in-vivo* phenotype was attributable to cell size. Collectively, the data suggest a role for Als1 in *C. albicans* cell size homeostasis, a novel hypothesis for further exploration.

## Introduction

Although most humans are colonized with *Candida albicans* as a commensal, disruptions in immune function or of the microbiota can lead to disease (Lopes and Lionakis, 2022). Understanding the host-fungus interaction at a mechanistic level is a main reason to study *C. albicans*. *In vitro* and *in vivo* phenotypic analysis of strains containing targeted mutations is a common approach to studying gene function (Noble et al., 2010). *C. albicans* encodes several gene families that are implicated in host-pathogen interactions (Butler et al., 2009). One example is the *ALS* (agglutinin-like sequence) family which includes eight genes that encode large cell-surface glycoproteins. Als protein function is most frequently discussed in terms of adhesive (i.e. ligand-binding) and aggregative interactions that mediate interaction of *C. albicans* with host cells, abiotic surfaces, other microbes, and even with each other (Hoyer and Cota, 2016).

Disruption of the first *ALS* gene, *ALS1*, reduces adhesion of *C. albicans* germ tubes to human vascular endothelial cells in *in vitro* assays (Fu et al., 2002; Zhao et al., 2004). Strains lacking *ALS1* also display delayed germ-tube formation *in vitro*. *In vivo* analyses using a murine model of disseminated candidiasis reveal a difference in hypha length of fungi in kidney sections when comparing the *als1/als1* strain to a wild-type control (Fu et al., 2002). When assessed at 28 h post-inoculation, mutant hyphae in a kidney section are obviously shorter than the control hyphae. However, by 40 h, the strains are indistinguishable. Survival analysis shows delayed disease progression for mice inoculated with the *als1/als1* strain, however, none of the mice in this experimental group survive. Comparisons between the *als1/als1* strain and wild-type control in a murine model of oropharyngeal candidiasis indicate a similar delay in disease progression: while the mutant strain lags behind the control on day 1 post-inoculation, the strains are indistinguishable by day 3 (Kamai et al., 2002).

*C. albicans* cultures for these experiments are often grown to saturation in yeast extract-peptone-dextrose medium (YPD; Fu et al., 2002; Zhao et al., 2004). Microscopic evaluation of a *C. albicans* strain lacking both *ALS1* alleles revealed that the strain has a greater proportion of smaller cells compared to cultures of a wild-type or *ALS1* reintegration control. The relationship between small cell size and delayed germ-tube formation is established in the literature (Chaffin and Sogin, 1976). These observations raised the question of whether phenotypic effects observed for strains lacking *ALS1* were attributable to a shift in the population toward a higher proportion of smaller cells. Several methods are used here to demonstrate that a *Δals1/Δals1* culture has a greater proportion of smaller cells compared to cultures of wild-type and *ALS1* reintegrant strains. Size selection of wild-type and mutant cells is employed to demonstrate the association between cell size and germ-tube-formation kinetics as well as disease progression in the murine model of disseminated candidiasis.

## Materials and Methods

### *C. albicans* Strains

*C. albicans* strain CAI12 (*iro1-ura3Δ*::*λimm^434^*/*IRO1 URA3*; Porta et al., 1999) was used as the control strain for all experiments. CAI12 was derived from the wild-type strain SC5314 (Gillum et al., 1984), and has a single copy of *URA3*, making it comparable to strains resulting from genetic manipulations with the *URA3* marker. Other strains used included 1467 (*iro1-ura3Δ*::*λimm^434^*/*iro1-ura3Δ*::*λimm^434^ als1_sa_Δ/als1_la_Δ-URA3*), and 2151 (*iro1-ura3Δ*::*λimm^434^*/*iro1-ura3Δ*::*λimm^434^ als1_sa_Δ/als1_la_Δ-ALS1_LA_-URA3)* (Zhao et al., 2004). In strains 1467 and 2151, *URA3* was located at the *ALS1* locus. The designations LA (large allele) and SA (small allele) distinguished between the two *ALS1* alleles from strain SC5314 that encoded different copy numbers of the 108-bp tandem repeat sequence within the central domain.

Strains 1690 and 1692 (*iro1-ura3Δ*::*λimm^434^*/*iro1-ura3Δ*::*λimm^434^ als1_sa_Δ/als1_la_Δ URA3*) were created to assess whether the *Δals1/Δals1* mutant phenotype could be attributed to placement of *URA3* in the *C. albicans* genome. To make these strains, strain 1468 (*iro1-ura3Δ*::*λimm^434^*/*iro1-ura3Δ*::*λimm^434^ als1_sa_Δ/als1_la_Δ ura3*) was transformed with a PCR fragment of approximately 3.5 kb that encoded the full *URA3* gene and a fragment that corrected the *IRO1* defect in strain CAI12. This fragment was amplified from SC5314 genomic DNA with primers URAupF (5’ GGTATGCACCTATAGATACTTGGA 3’) and URAdnR (5’ TGACTATAGTCTACCTATAACCTC 3’). The fragment was amplified with *Pfu* polymerase (Stratagene) and cloned into pCRBlunt (Invitrogen). The fragment was excised from the cloning vector with *Sst*I and *Xho*I and transformed into strain 1468 spheroplasts using published methods (Zhao et al., 2004). Transformants were selected on SC-Uri plates (Zhao et al., 2004) and checked for accuracy on Southern blots using the *URA3* coding region as a probe.

### Cell Culture

Strains were stored in 38% glycerol at –80°C. Cells from the glycerol stock were streaked onto a YPD agar plate (per liter: 10 g yeast extract, 20 g peptone, 20 g dextrose with 20 g Bacto agar for plates) and incubated for 24 h at 37°C. This plate was stored at 4°C for no more than one week. Colony size for all strains was uniform, consistent with a similar growth rate for each. One representative colony from the stock YPD plate was inoculated into 20 ml YPD medium in a 50-ml Erlenmeyer flask. The flask was incubated for 16 h at 37°C with 200 rpm shaking. These growth conditions resulted in a saturated culture (approximately 3 x 10^8^ cells/ml). Cultures were checked microscopically to confirm the lack of germ tubes and the presence of mainly single yeast cells (i.e. a low percentage of budding forms). The cells were collected by filtration through a sterile 0.45 μm pore-size nylon filter (Magna, GE Osmonics, Fisher Scientific). The filter was washed once with 20 ml Dulbecco’s phosphate-buffered saline without Ca^2+^ or Mg^2+^ (DPBS; Lonza catalog number 17-512Q) to remove associated growth medium from the cells. The nylon filter with the cells attached was transferred using sterile technique to a 50-ml conical polypropylene centrifuge tube. Six ml of DPBS were added to the tube and the tube and filter vortexed to remove cells from the filter. The DPBS was removed to a sterile 50-ml conical tube and the washing procedure repeated two more times. All three washes were pooled giving a volume approximately equal to the initial culture volume. For flow cytometry analysis or cell sorting, the washed cell stock was diluted 1:20 in sterile DPBS.

### Coulter Counter Analysis

The Coulter Counter (Model ZM, Coulter Electronics Ltd.) was fitted with a 30 µm orifice tube and calibrated using 2 different certified latex particles of known size (2 and 5 μm). The half-count method was used to identify the median size (bead volume) value (Coulter Electronics Ltd., 1985). The calibration constant obtained was automatically calculated by the instrument and used in all measurements performed with a given lot of electrolyte solution (cell diluent). The instrument was recalibrated each time a new electrolyte solution was prepared. Typical instrument settings used were a lower threshold of 53 and an upper threshold of 99.9; attenuation was set at 256 and aperture current was 250 mA. All calibration and experimental measurements of particle sizes were made under constant gentle stirring to maintain the particles or yeast cells in a uniform suspension. Experiments were conducted in duplicate on a given day and the mean cell volume measurements graphed using Microsoft Excel software. Experiments were conducted on cultures grown independently on at least three separate days to ensure consistency of results.

### Manual Measurement of *C. albicans* Cell Size

Cells were placed onto glass microscope slides and examined using a Nikon Eclipse E600 microscope equipped with a digital camera (QImaging; Teledyne Photometrics, Tucson, AZ, USA). Images of cells at 400× magnification were captured and stored. Images were opened in Adobe Photoshop software and enlarged using a standardized method so cells could be measured using a millimeter ruler on a printed page. Mean cell diameter was calculated as the average of the longest and shortest ‘diameter’ (measured at right angles to each other) for each cell (Soll et al., 1981). Measurements from printed images were made to the nearest 0.5 mm. Using the same protocol as for yeast cells, Polystyrene Uniform Microspheres (Bangs Laboratories Inc., Fishers, IN, USA) of known diameter were visualized microscopically, imaged, and measured by hand. This information was used to convert yeast cell measurements to microns. One hundred yeast cells were measured for each culture on each day of the experiment. The experiment was completed on three separate days to ensure consistent results.

### Flow Cytometry Methods

Cell sorting and flow cytometry analysis of scatter properties used a MoFlo instrument (Cytomation Inc., Fort Collins, CO, USA). The cells were illuminated with a 488 nm laser line at 100 mW. Gating on area versus width of forward angle light scatter signal was applied to exclude doublets (budding cells). The cells were sorted at 500 events/sec at room temperature. Sorted cell populations were collected using a 0.45-μm-pore-size filter (Magna, GE Osmonics, Fisher Scientific). Cells were washed from the nylon filter using 1 ml DPBS and quantified using a hemacytometer.

### Germ-Tube-Formation Assays

A total of 5 x 10^5^ cells was added to 3 ml of one of the following prewarmed germ-tube-inducing media: RPMI 1640 with L-glutamine (Invitrogen catalog number 11875), Lee medium (Lee et al., 1975), YPD with 10% fetal bovine serum, or Spider medium (Liu et al., 1994) in a 25-ml Erlenmeyer flask. Culture flasks were incubated at 37°C with 200 rpm shaking for various times (specified in Results for each experiment). Cultured cells were harvested by vacuum filtration onto 1 μm pore-size filters (Whatman polycarbonate Nucleopore membranes, Fisher Scientific) and 5 ml of DPBS used to wash away residual medium. Filters were removed from the vacuum filtration unit, inverted onto glass slides and dried. Following removal of the filter, slides were heat fixed and then stained with crystal violet for 2 min. The slides were washed with tap water and dried. Slides were examined microscopically. Cells with a germ tube at least as long as the diameter of the mother yeast were considered positive. Five random fields were examined for a total of 250 to 300 individual cells for each culture condition. The mean and standard deviation from duplicate experiments from the same day were reported. Duplicate experiments were conducted on one or more additional days to verify that consistent results were observed.

### Murine Model of Disseminated Candidiasis

Female BALB/cByJ mice (7 weeks old) were purchased from The Jackson Laboratory (Bar Harbor, ME, USA). *C. albicans* yeast cells were prepared using methods described above for culture growth and, when indicated, cell sorting. Inoculum sizes are specified for each experiment in Results. A constant volume of 0.1 ml was used for each mouse and injected into the lateral tail vein. A group size of 5 mice was used. The University of Illinois Institutional Animal Care and Use Committee approved all experimental animal protocols. At the specified time points, mice were euthanized and the kidneys removed. Tissue for measuring cfu/g was weighed, homogenized in DPBS, and serial dilutions plated on YPD agar. Colonies were counted following 24 h incubation at 37°C. Data were analyzed with the Mann-Whitney test (GraphPad Prism Software, San Diego, CA, USA). Comparisons with P < 0.05 were considered significant. Tissue for histopathology was fixed for 24 h in 10% neutral buffered formalin, trimmed, embedded in paraffin, and sectioned. Sections were stained with Gomori metheneamine silver (GMS).

## Results

### Deletion of *ALS1* Increased the Proportion of Smaller Cells in a Culture-Flask Population

**Figure 1:**
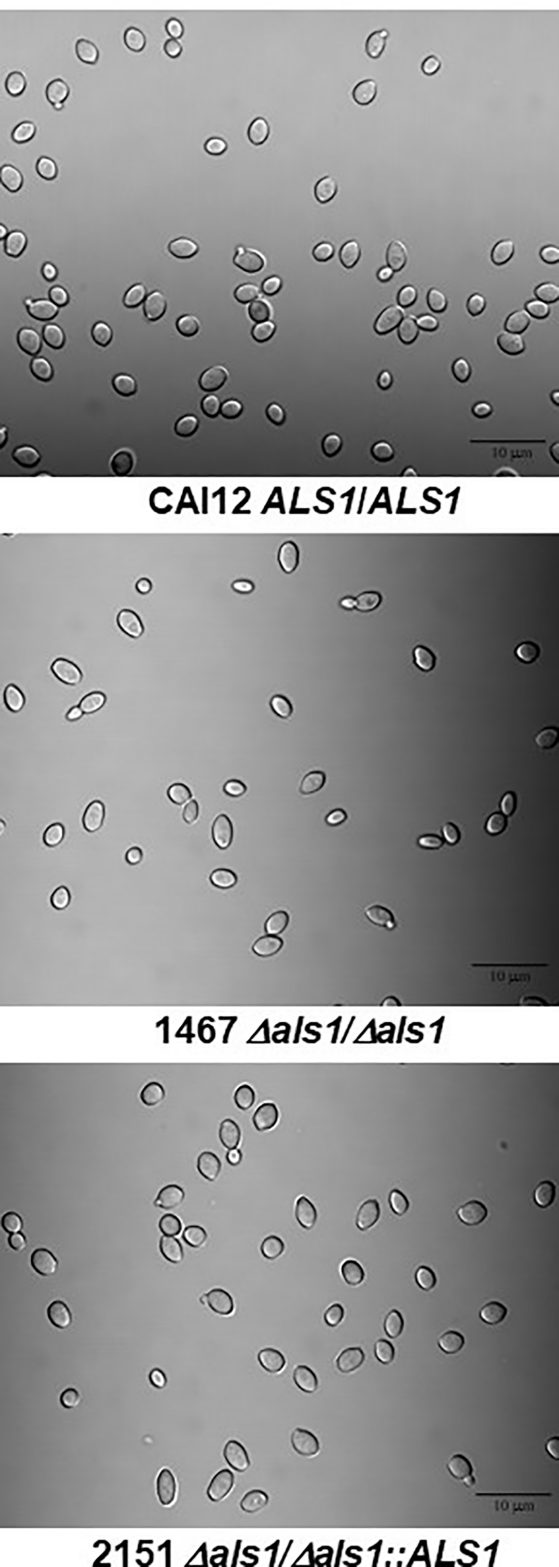
Light micrographs to demonstrate cell sizes present in YPD cultures of *C. albicans* strains. *C. albicans* were grown for 16 h in YPD medium at 37°C and 200 rpm shaking then visualized microscopically (400× magnification). The micrographs show the range of cell sizes present in each culture. Overall, the culture of strain 1467 (*Δals1/Δals1*) contained a greater proportion of smaller-sized cells than the control strain CAI12 or the reintegrant strain 2151 (*Δals1/Δals1::ALS1*). The scale bar in each image indicates 10 microns.

Growth of *C. albicans* strains in the rich medium YPD is a standard method for preparing cells for phenotypic characterization and was the only method reported in studies that characterized the germ-tube formation and virulence phenotypes of *als1/als1* strains (Fu et al., 2002; Zhao et al., 2004). Microscopic evaluation suggested that, when grown to saturation in YPD, the culture population for strain 1467 (*Δals1/Δals1*) had proportionally more smaller cells than the CAI12 control (**Figure 1**). Examination of a strain into which a wild-type *ALS1* allele was reintegrated (strain 2151; *Δals1/Δals1::ALS1*) suggested that the population distribution of cell sizes shifted back toward the CAI12 control. Previous work showed that there was no significant difference between doubling times for strains CAI12 (1.28 ± 0.09 h), 1467 (1.33 ± 0.09 h), and 2151 (1.30 ± 0.09 h) (Zhao et al., 2004).

**Figure 2:**
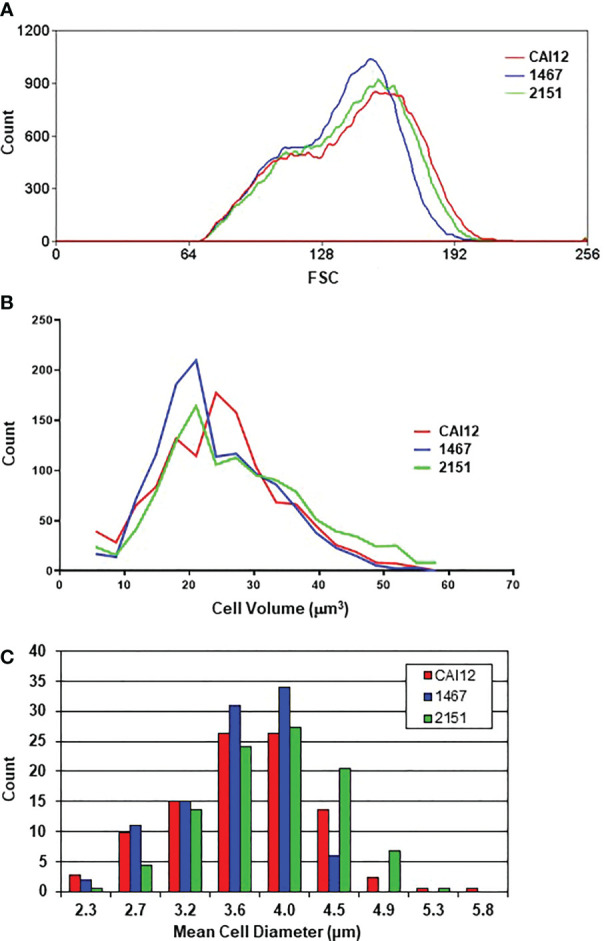
Distributions of cell sizes in YPD cultures of *C. albicans* CAI12, 1467, and 2151 evaluated using various methods. Cell-size distributions were measured using flow cytometry **(A)**, a Coulter Counter **(B)**, and manually from printed light micrographs **(C)**. Results from each method showed the overall leftward shift of the distribution for strain 1467 (*Δals1/Δals1*), consistent with the conclusion that the strain produced a higher proportion of smaller-sized cells compared to either strain CAI12 or 2151.

Although the distribution of cell sizes and their relationships among the strains were visible in light micrographs (**Figure 1**), additional methodologies were used to describe these differences among the cultured-cell populations. Culture populations of strains CAI12, 1467, and 2151 were analyzed by flow cytometry, measuring forward angle light scatter (FSC; **Figure 2A**). The population of strain 1467 cells (*Δals1/Δals1*) showed a leftward shift compared to the control strain CAI12 and *ALS1* reintegrant strain 2151. Coulter Counter analysis, which measured cellular volume, produced a similar leftward-shifted population for strain 1467 with notably more *Δals1/Δals1* cells in the range of 15-20 μm^3^ compared to the others (**Figure 2B**). Cells hand measured from printed micrographs also showed the leftward shift for strain 1467 relative to the control strains, indicating a greater abundance of smaller cells in the strain 1467 culture than for CAI12 or 2151 (**Figure 2C**). These results validated flow cytometry as a method to describe cell-size distributions in *C. albicans* populations and convincingly demonstrated that *ALS1* deletion increased the relative proportion of smaller cells in the culture-flask population.

### Delayed *Δals1/Δals1* Germ-Tube Formation in All Growth Conditions Tested

Chaffin and Sogin (1976) studied the relationship between *C. albicans* cell size and germ-tube-formation kinetics using Lee medium. The authors showed that smaller *C. albicans* cells were delayed in germ-tube formation. A defect in germ-tube formation for a *C. albicans* strain lacking *ALS1* was first noted on solid and in liquid Lee medium (Fu et al., 2002). Subsequent work suggested a possible growth-medium-specific effect because the germ-tube-formation defect was not apparent in RPMI 1640 medium at 60 min (Zhao et al., 2004). The relationship between germ-tube formation for strain 1467 and the CAI12 and 2151 control strains was revisited using a kinetic approach and multiple culture conditions that promoted germ-tube formation.

**Figure 3:**
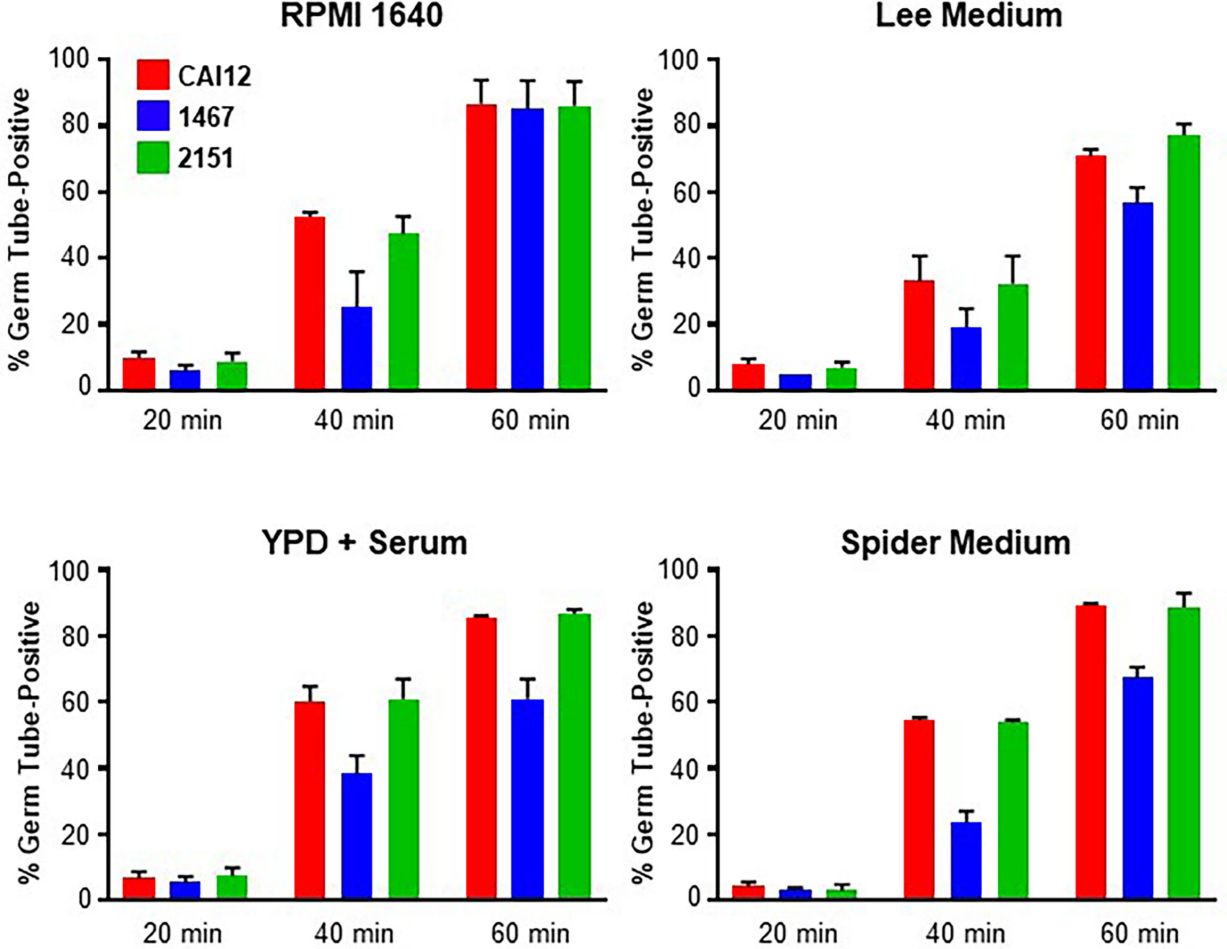
Histograms showing percent positive *C. albicans* cells in a germ-tube-formation assay. *C. albicans* CAI12, 1467, and 2151 were grown to saturation in YPD, washed and processed as described in Materials and Methods. Cells were inoculated into four different growth media that induced germ-tube formation. Cells were assessed every 20 min to count those that had a germ tube length greater than or equal to the diameter of the mother yeast. The mean and standard deviation for duplicate assays are shown. In each growth medium, germ-tube formation for strain 1467 (*Δals1/Δals1*) lagged behind that for the control (CAI12) and reintegrant (2151) strains. The assays were repeated in duplicate on one or more additional days to verify the lag in strain 1467 germ-tube formation and to establish 40 min as a time point where the lag could be observed.

Strains CAI12, 1467, and 2151 were grown to saturation in YPD medium using the conditions described above for the cell-size measurement experiments. The *C. albicans* cells were then transferred to one of four different germ-tube-induction media: RPMI 1640, Lee medium, YPD + 10% fetal bovine serum, or Spider medium. Percent positive cells were counted at 20-, 40-, and 60-min time points. Positive cells were those with a germ tube equal to or longer than the diameter of the mother yeast. A similar percentage of cells with germ tubes was observed for each strain at the 20-min time point in each growth medium (**Figure 3**). By 40 min, however, differences between the control (CAI12 and 2151) and *Δals1/Δals1* strain 1467 were obvious. At 60 min in RPMI 1640 medium, germ-tube formation for strain 1467 caught up to the control. In the other media, germ-tube formation took a longer time to reach the percentage exhibited by the controls. Microscopic evaluation of the cultures showed that mother yeasts were forming germ tubes for strain 1467, but the germ tubes were shorter than for the other strains. The kinetic approach to the germ tube assays reconciled the previous seemingly disparate conclusions about the effect of *ALS1* deletion on germ-tube formation. These results demonstrated a general defect in germ-tube formation for strain 1467 (*Δals1/Δals1*) that was not growth-medium-specific. The work also established the 40-min time point for use in all subsequent assays.

### Use of Cell Sorting to Demonstrate the Effect of Cell Size on Germ-Tube Formation in *ALS1/ALS1* Strain CAI12

Experiments above showed that a culture of strain 1467 (*Δals1/Δals1*) had a greater proportion of smaller cells than cultures of strains CAI12 and 2151 and that strain 1467 lagged behind the control strains in germ-tube formation. Cell sorting was used to create populations of different-sized yeast cells to further establish these relationships in the wild-type and mutant strains.

**Figure 4:**
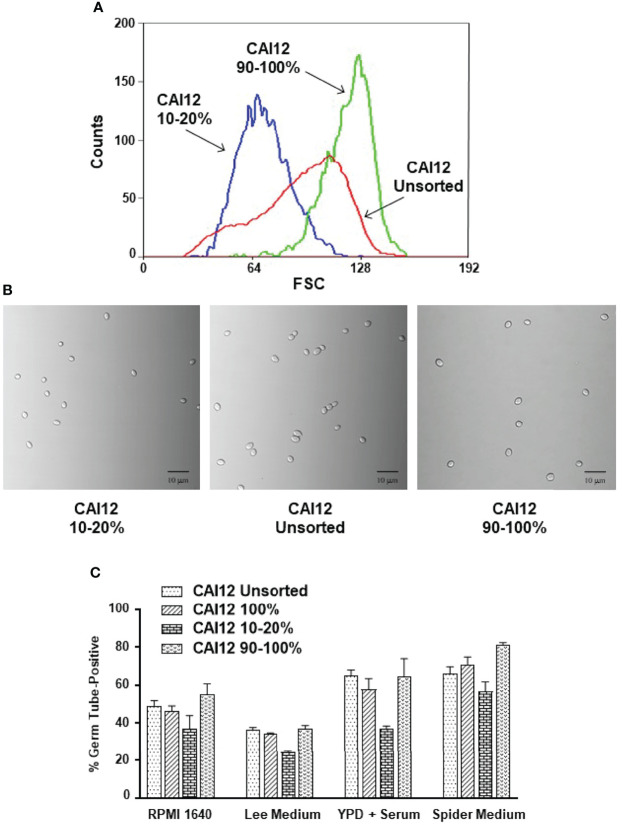
Use of flow cytometry to select *C. albicans* CAI12 (control) cell populations of varying size, and subsequent testing of the populations in a germ-tube-formation assay. **(A)** Histogram of CAI12 cultured cell population distributions showing the relationship between an unsorted culture and CAI12 populations for which the largest 10% (called 90-100%) and a 10% fraction of small cells (called 10-20%) were collected. **(B)** Light micrographs of *C. albicans* cells from each of the CAI12 populations to show the sizes of cells collected. The scale bar in each image indicates 10 microns. **(C)** Histogram showing the results of a germ-tube-formation assay using the CAI12 cell populations derived using flow cytometry. The unsorted, 90-100%, and 10-20% populations were described and shown above. The population labeled 100% was the unsorted population that was run through the flow cytometer and collected. This control evaluated whether the flow sorting process affected formation of germ tubes. Following flow cytometry, CAI12 cells were collected by filtration, counted using a hemacytometer, and inoculated into various culture media that promoted germ-tube formation. A 40-min time point was used. Duplicate assays were conducted and the mean and standard deviation shown. The data shown are representative of duplicate assays on two separate days. Results clearly demonstrated that selecting smaller (wild-type control; *ALS1/ALS1*) cells resulted in delayed germ-tube formation even though the cells were identical genetically to the other populations.

A CAI12 population was sorted to select small cells (from the left side of the distribution; called CAI12 10-20%) and the largest 10% of the cells (called CAI12 90-100%; **Figure 4A**). Light micrographs showed the populations of CAI12 cells resulting from this procedure, compared to the starting culture of unsorted cells (**Figure 4B**). The entire CAI12 cell population was passed through the flow cytometer to control for the effect of the sorting process on germ-tube formation; these cells were called “CAI12 100%” (**Figure 4C****)**. The close relationship between results for the unsorted control and the 100% group suggested that the sorting process did not affect germ-tube formation. Smaller cells (CAI12 10-20%) clearly lagged behind the control in germ-tube formation, while the largest cells (CAI12 90-100%) tended to match or exceed the percent positive cells observed in the control samples. These data demonstrated how manipulation of cell size distribution in a population affected conclusions about germ-tube formation in a wild-type strain.

### Redistribution of *Δals1/Δals1* Population Cell Sizes Produced Wild-Type Germ-Tube-Formation Kinetics

**Figure 5:**
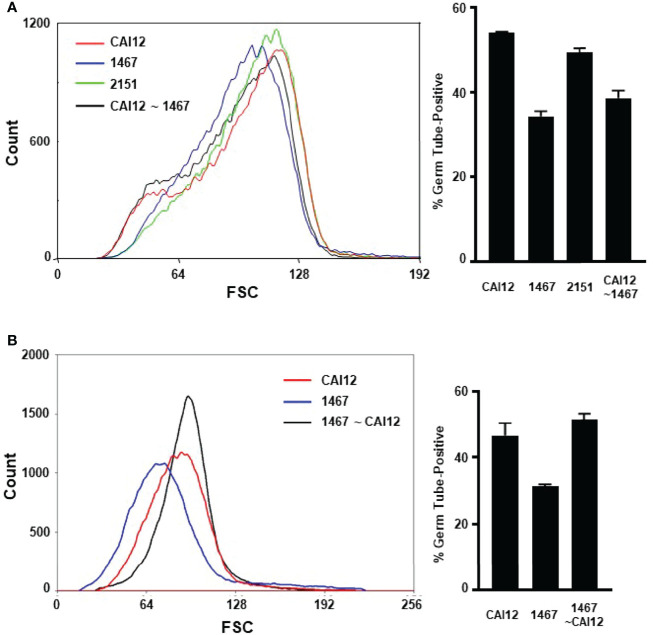
Use of flow cytometry to redistribute cultured cell populations to mimic the germ-tube-formation kinetics of different genetic backgrounds. **(A)** Flow cytometry was used to redistribute the population of CAI12 cells to more-closely approximate the 1467 (*Δals1/Δals1*) population distribution. The sorted population was called CAI12 ~ 1467 (black line; graph at left). The distribution of sorted cells was compared to unsorted populations of control cells (CAI12), as well as 1467 (*Δals1/Δals1*), and 2151 cultures (*Δals1/Δals1::ALS1*; left). Sorting CAI12 to select smaller cells shifted its distribution (originally the red line) toward the 1467 distribution (blue). Cells from all four populations were placed into RPMI 1640 medium for 40 min and germ-tube formation evaluated. Redistribution of the CAI12 culture into the CAI12 ~ 1467 population enriched for smaller cells, which had delayed germ-tube-formation kinetics. Therefore, the wild-type strain was manipulated to appear like the mutant strain by mimicking its cell-size distribution. **(B)** The converse cell redistribution experiment sorted the 1467 population (*Δals1/Δals1*; blue line at left) to more-closely resemble the CAI12 population (red line). The new population was called 1467 ~ CAI12 (black line). Enrichment of larger cells in the 1467 ~ CAI12 population resulted in germ-tube-formation kinetics that were similar to the control strain (CAI12). Because mutant cells were able to form germ tubes at the same rate as the control strain, the altered cell-size distribution for the *Δals1/Δals1* strain was responsible for its apparent defect in germ-tube formation exhibited by the unsorted culture. Assays were conducted in duplicate and the mean and standard deviation shown. The data shown were representative of duplicate assays on two separate days.

Data from the cell-sorting experiment suggested that the germ-tube-formation kinetics of a culture flask population represented the aggregate of germ-tube-formation rates of the various cell sizes present. If this concept was correct, redistributing the population size of wild-type cells to match the strain 1467 distribution should result in germ-tube-formation percentages similar to the mutant strain. CAI12 cells were sorted so the population more-closely resembled the size distribution of the 1467 culture (**Figure 5A**). Germ-tube formation was tested in RPMI 1640 medium. As predicted, shifting the CAI12 size distribution to the left reduced the percent germ-tube-positive cells to nearly the strain 1467 level.

The reverse approach to the experiment was also tried by sorting the strain 1467 population to more-closely resemble the CAI12 culture (**Figure 5B**). Selecting the larger cells from the 1467 culture resulted in an increase in germ-tube-positive cells in the assay. These results demonstrated that cells in which *ALS1* was deleted could form germ tubes like a wild-type strain if cell size was normalized between the populations. The sorting process involved in these experiments also reinforced the differences in cell-size distribution between the CAI12 and 1467 culture populations. For example, it was easier to sort CAI12 to mimic 1467 than to sort 1467 to mimic CAI12. The latter sorting process would be simpler if the 1467 culture had a greater proportion of larger cells, but it did not. These experiments convincingly demonstrated that cell size, rather than another effect of *ALS1* deletion, was the main factor responsible for delayed *in vitro* germ-tube formation of the *Δals1/Δals1* strain.

### Heading *in vivo*: The Need to Examine *URA3* Positional Effects

Gene deletions created using the *URA3* selectable marker can display altered phenotypes in various assays due to positional effects of the gene (reviewed in Staab and Sundstrom, 2003). In strain 1467 (Zhao et al., 2004) and the other published *als1/als1* strain (Fu et al., 2002), the *URA3* marker was located in the *ALS1* locus on chromosome 6 rather than at its native locus on chromosome 3. To determine whether positional effects of *URA3* affected experimental results, the *URA3* gene was removed from the *ALS1* locus and placed at its native locus. This approach yielded strains 1690 and 1692, two independent transformants from the construction procedure.

**Figure 6:**
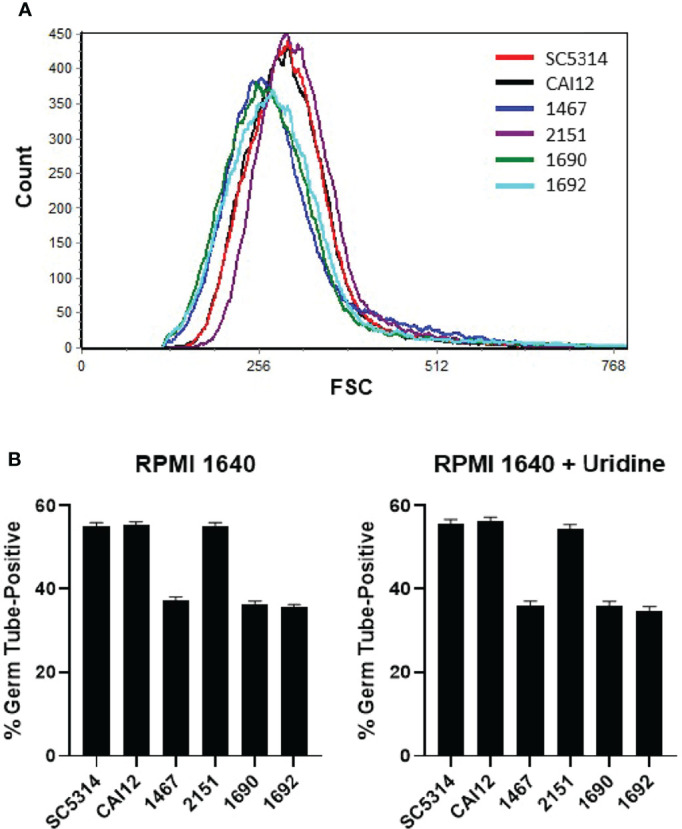
Flow cytometry and germ-tube-formation assays to evaluate the effect of *URA3* on *C. albicans* phenotype. **(A)** Histogram of *C. albicans* cell populations comparing control strains (SC5314 and CAI12) to *Δals1/Δals1* strains (1467, 1690, 1692), to the *Δals1/Δals1::ALS1* strain 2151. Strain 1467 differed from 1690 and 1692 because *URA3* was placed at the *ALS1* locus in 1467 and at the *URA3* locus in 1690 and 1692. All the *Δals1/Δals1* strains showed the leftward shift associated with cell populations that included a greater abundance of smaller cells. These results demonstrated that placement of the *URA3* marker did not affect the cell distribution of the *Δals1/Δals1* strains. **(B)** When placed into RPMI 1640 medium for 40 min, all *Δals1/Δals1* strains showed delayed germ-tube formation. This delay was not rescued by addition of exogenous uridine, suggesting that effects of *ALS1* deletion on cell size and germ-tube formation were not due to placement of the *URA3* marker in the *C. albicans* genome. Assays were conducted in duplicate. The mean and standard deviation are shown. Data shown were representative of duplicate assays on two separate days.

Flow cytometry analysis of these strains showed a leftward shift of the cell-size distribution, similar to strain 1467 (**Figure 6A**). This leftward shift was an obvious contrast to distributions for strains CAI12, 2151, and parent strain SC5314. The favorable comparison between strains SC5314 (two *URA3* copies) and CAI12 (one *URA3* copy) demonstrated that cell-size distributions were not affected by *URA3* dosage.

Germ-tube formation for the *Δals1/Δals1* strains did not differ regardless of the locus where *URA3* was located (**Figure 6B**). Addition of exogenous uridine (25 μg/ml) to the germ-tube-induction medium also did not affect the results. These data demonstrated clearly that the cell-size effects and germ-tube-formation kinetics observed for strain 1467 were not caused by the position of the *URA3* marker.

### Evaluating the Relationship Between *C. albicans* Cell Size and Disease Progression in a Murine Model of Disseminated Candidiasis

**Figure 7:**
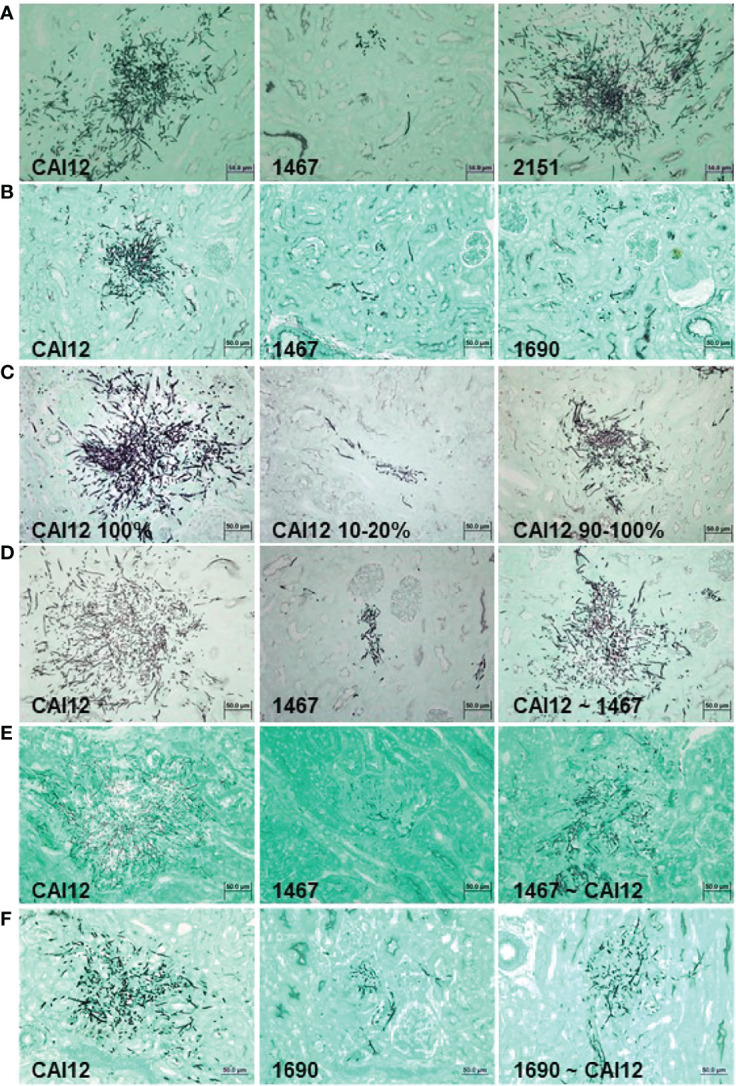
Light micrographs of GMS-stained kidney sections from mice that were intravenously inoculated with *C. albicans* to assess relative virulence of *C. albicans* populations. Experimental detail for each row of images **(A–F)** is provided in **Table 1**. Populations for which there was a greater proportion of larger cells showed more-extensive growth in the mouse kidney. Relative virulence of the control strain (CAI12; *ALS1/ALS1*) was reduced by sorting a population of smaller cells from the culture, while selecting a population of larger cells from an *Δals1/Δals1* strain increased relative virulence. These results demonstrated that the cell-size defect in the *Δals1/Δals1* strain had an obvious effect on virulence in the murine model. The scale bar in each image indicates 50 microns.

Previous testing of an *als1/als1* strain in a murine model of disseminated candidiasis showed delayed virulence at 28 h that, by 40 h, caught up to a control strain (Fu et al., 2002). Histopathology of kidney lesions showed far fewer *C. albicans als1/als1* cells compared to the control strain. Hypha formation was also reduced *in vivo* for the *als1/als1* strain. *In vitro* results presented here suggested that cell-size variation was responsible for germ-tube-formation differences between strains 1467 (*Δals1/Δals1*) and control strains prompting similar testing of cells in the murine model. **Table 1** summarizes results from those experiments with corresponding histopathology images presented in **Figure 7**.

**Table 1:** Virulence assessments using the murine model of disseminated candidiasis.

Strain	Inoculum^*^ (no. of cells)	Time point (h)	log_10_ ± SD cfu/g kidney	Image row in Figure 7
CAI12	8 x 10^5^	28	5.61 ± 0.09	
1467			4.03 ± 0.26^†^	A
2151			5.92 ± 0.47^§^	
CAI12	5 x 10^5^	28	5.53 ± 0.33	
1467			4.14 ± 0.10^†^	B
1690			4.66 ± 0.21^†§^	
CAI12 (100%)	1 x 10^6^	28	5.26 ± 0.13	
CAI12 (90-100%)			5.58 ± 0.34^§^	
CAI12 (10-20%)			4.72 ± 0.21^†^	C
CAI12 (10-20%)		48	5.27 ± 0.08	
CAI12	1 x 10^6^	28	5.76 ± 0.28	
1467			4.77 ± 0.26^†^	Not shown
CAI12 ~1467			5.75 ± 0.08^§^	
CAI12	4 x 10^5^	28	5.28 ± 0.05	
1467			3.85 ± 0.17^†^	D
CAI12 ~ 1467			4.98 ± 0.38^§^	
CAI12	5 x 10^5^	28	6.16 ± 0.14	
1467			4.99 ± 0.27^†^	E
1467 ~ CAI12			5.59 ± 0.12^†§^	
CAI12	5 x 10^5^	28	5.90 ± 0.06	
1690			4.84 ± 0.26^†^	F
1690 ~ CAI12			5.76 ± 0.11^†§^	

^*^ Fu et al. (2002) used an inoculum size of 1 x 10^6^ cells and a 28-h time point for virulence assessments in the murine model of disseminated candidiasis. Inoculum sizes selected here were within that range yet decreased to accommodate the time-intensive nature of collecting sorted cells. Sorted cells for inoculating mice were also used for germ-tube-formation assessments, necessitating a smaller inoculum size to complete all the experiments on the same day. The relationship between CAI12 (ALS1/ALS1) and 1467 (Δals1/Δals1) was significantly different in each experiment, regardless of inoculum size as described.

^†^ Significantly different from the CAI12 control or from CAI12 (100%) (P < 0.05).

^§^ Significantly different in another comparison detailed in the text (P < 0.05).

Inoculation of mice with 8 x 10^5^ C*. albicans* cells of strains CAI12, 1467, or 2151 showed a significant decrease in cfu/g kidney for the *Δals1/Δals1* strain (1467) compared to the CAI12 control. Reintegration of a wild-type *ALS1* allele (strain 2151) complemented the defect in the mutant strain. Histopathology images of kidney tissue showed greatly decreased fungal colony sizes, with a large reduction in hypha formation for strain 1467 compared to the controls (**Figure 7A**). These data reproduced the results reported by Fu et al. (2002) using strains described here.

Strain 1690, in which *ALS1* was deleted and the *URA3* gene integrated at its own locus, was also tested to explore whether placement of the *URA3* gene affected the virulence phenotype of the mutant strain. Both strain 1467 and 1690 showed a significant decrease in cfu/g kidney compared to the CAI12 control (**Table 1**). Additionally, strain 1690 significantly differed from strain 1467, demonstrating an effect of *URA3* location on *Δals1/Δals1* virulence. This difference was visible in histopathology images that showed a greater abundance of strain 1690 cells in the mouse kidney compared to strain 1467. Both *Δals1/Δals1* strains showed an overall decrease in hypha length compared to the CAI12 control (**Figure 7B**).

CAI12 cells were sorted to isolate a small-cell fraction (10-20%; **Figure 4B**) and a 90-100% fraction, to assess the effect of *ALS1/ALS1* cell size on virulence. The entire CAI12 culture was passed through the cell sorter (100%) and used as a control. Mice inoculated with the CAI12 10-20% cells showed a significantly decreased cfu/g kidney tissue compared to the 100% or 90-100% populations (**Table 1**). Over time, cfu/g for the 10-20% mice increased, consistent with the delayed virulence phenotype described previously for the *als1/als1* strain (Fu et al., 2002). Histopathology images showed fewer and smaller lesions for the 10-20% cells compared to the 100% sample (**Figure 7C**). The 90-100% cells produced smaller, but more numerous, lesions than the 100% cells. These data demonstrated that the distribution of cell sizes affected kidney cfu/g as well as the character of the resulting lesions.

CAI12 and 1467 populations were also sorted to redistribute cell sizes. In the first experiment, CAI12 was sorted to resemble the 1467 distribution (similar to **Figure 5A**; called CAI12 ~ 1467). Two different inoculum doses were tested (**Table 1**). For each, there was no significant difference between cfu/g for the sorted and unsorted CAI12 cells. However, histopathology images showed reduced fungal colony size in the kidneys for the sorted population (**Figure 7D**). For these cells, sorting CAI12 to resemble the 1467 population distribution decreased germ-tube formation *in vitro*, but did not decrease cfu/g *in vivo* despite the reduced fungal colony size.

The experiment was conducted from the converse perspective, sorting the strain 1467 culture to more-closely resemble the CAI12 distribution (called 1467 ~ CAI12). Because the 1467 culture did not have as many large-sized cells as the CAI12 culture, it was more difficult to get the population distributions to match as when sorting CAI12 cells. However, sorting achieved an enrichment of larger 1467 (*Δals1/Δals1*) cells in the population (similar to **Figure 5B**). When inoculated into mice, there was a significant increase in cfu/g for the 1467 ~ CAI12 cells compared to the 1467 culture (P = 0.008). However, the CAI12 cfu/g was still higher than 1467 ~ CAI12 (P = 0.04). Histopathology images showed increased lesion size for the 1467 ~ CAI12 cells and showed lesions that almost equaled CAI12 lesions in size and hypha length (**Figure 7E**). Overall, lesions in the 1467 ~ CAI12 mice were less abundant than for the CAI12 mice.

Similar results were observed for sorting the 1690 population to enrich for larger cells (**Table 1**; **Figure 7F**). Although fungal-burden measurements for 1690 ~ CAI12 cells were quite close to the CAI12 control, the two groups were still significantly different (P = 0.03). Compared to strain 1467, placement of *URA3* at its own locus in strain 1690 produced fungal burden that more-closely approximated the control. Results for strains 1467 and 1690 could possibly be pushed to statistical significance by using larger groups of mice. However, the smaller group size used here required fewer sorted cells, which permitted high-quality experiments and timely completion of the sorting process. Collectively, the results demonstrated that cell size, rather than another effect of *ALS1* deletion, was the main factor responsible for the delayed disease progression phenotype of the *Δals1/Δals1* strain.

## Discussion

In previous work, delayed germ-tube formation *in vitro*, smaller germ tubes in the mouse kidney, and delayed disease progression in the mouse model led to the suggestion that Als1 functions in initiation of germ-tube formation (Fu et al., 2002). Data presented here show that delayed germ-tube formation (both *in vitro* and *in vivo*) and delayed disease progression in the murine model are largely due to the effects of *ALS1* deletion on *C. albicans* cell size. These data suggest that Als1 functions in cell size homeostasis, rather than initiation of germ-tube formation.

Research to understand the coordination of eukaryotic cell growth and division has revealed many conserved themes in evolutionarily diverse species (Murray and Hunt, 1993). *Saccharomyces cerevisiae* is one model organism for these studies; many yeast cell-division processes are shared between *S. cerevisiae* and *C. albicans*. Research effort also has been dedicated to determining the *C. albicans* cell cycle processes that function during hypha and pseudohypha growth (reviewed in Sudbery et al., 2004). Two cell-size checkpoints are present during mitotic growth of yeast forms: one at Start (late G1) and the other at the G2/M transition. Once cells pass Start, they are committed to mitosis (Hartwell and Unger, 1977). Passing Start prematurely leads to cells smaller than wild-type, while a delay in passing Start leads to cells larger than wild-type. At the G2/M checkpoint, premature entry into mitosis results in a mother cell of the wild-type size and an abnormally small daughter cell (Kellogg, 2003). Delay at the G2/M checkpoint results in mother and daughter cells that are the same size, rather than a daughter cell that is slightly smaller than the mother as in wild-type cells (Hartwell and Unger, 1977; Kellogg, 2003). Viable cells with altered size (either larger or smaller than wild-type) arise from mutations in genes at either checkpoint (Jorgensen et al., 2002; Zhang et al., 2002).

We hypothesize that *ALS1* deletion results in premature mitotic entry (shortened G2/M transition) and generation of smaller daughter cells. The mechanism by which cells sense an appropriate critical size during the cell division process is an area of active investigation. Data presented here suggest that Als1 may contribute to the cell-size-sensing mechanism in *C. albicans*. Als1 is most commonly discussed in the context of binding host-cell ligands, an activity mediated by a peptide-binding cavity in the N-terminal domain of the molecule (Salgado et al., 2011; Lin et al., 2014). Als proteins bind a wide variety of ligands, suggesting the potential for contact with other *C. albicans* surface proteins (reviewed in Hoyer and Cota, 2016). Als proteins also have an amyloid-forming region that promotes aggregation among proteins on the same cell, as well as proteins on different cells (Lipke et al., 2012). It is easy to envision Als1 aggregative activity contributing to sensing of cell size. *C. albicans* Als3 strains with mutations that destroy peptide-binding-cavity-mediated adhesion or amyoid-forming-region-mediated aggregation were created and promoted understanding of the contributions of each function to *C. albicans*-host cell interactions (reviewed in Hoyer and Cota, 2016). Perhaps similar mutations in Als1 would promote the leftward shift in cell-size distribution displayed here for the *Δals1/Δals1* deletion strain, or perhaps such strains would rule out contribution of the peptide-binding cavity or amyloid-forming region to cell-size effects.

The new information presented here suggests that cell size should be evaluated for *C. albicans* mutant strains that exhibit the phenotype of delayed germ-tube formation *in vitro* and *in vivo*, as well as delayed disease progression. These phenotypes could be the result of the shifted size distribution demonstrated for deletion of *ALS1*. Methods detailed here provide tools for evaluating cell-size distribution and its contribution to *C. albicans* phenotype.

## Data Availability Statement

The raw data supporting the conclusions of this article will be made available by the authors, without undue reservation.

## Ethics Statement

The animal study was reviewed and approved by University of Illinois at Urbana-Champaign Institutional Animal Care and Use Committee.

## Author Contributions

LH conceptualized the study, acquired funding, administered the project, and wrote the original manuscript draft. XZ, S-HO, DC, and LH developed the study methodology, performed the investigation, and conducted formal analysis. All authors contributed to the article and approved the submitted version.

## Funding

This research was funded by Public Health Service grant R01 DE14158 from the National Institute of Dental and Craniofacial Research, National Institutes of Health.

## Conflict of Interest

The authors declare that the research was conducted in the absence of any commercial or financial relationships that could be construed as a potential conflict of interest.

## Publisher’s Note

All claims expressed in this article are solely those of the authors and do not necessarily represent those of their affiliated organizations, or those of the publisher, the editors and the reviewers. Any product that may be evaluated in this article, or claim that may be made by its manufacturer, is not guaranteed or endorsed by the publisher.
